# On Prof. Armor’s Prize Essay

**Published:** 1853-11

**Authors:** 


### *Professor Armor's Prize Essay.*

We regret very much our inability to publish entire this valuable essay. In accordance with our design, we will proceed to give a condensed abstract from it, in order that our readers may see the design, and the importance of this treatise,—the result of so much investigation and research.

Waiving all discussion with regard to the independent vitality of the blood, the manner of its development, growth or decay, its purposes and its uses, whether for nutrition, or to supply the wants and demands of the various structures by the elaboration of fibrine; the author avoids these discussions, we say, and proceeds to the investigation into the presence of foreign matters in that fluid, in our essential or Idiopathic forms of fever. That there is an admixture of deleterious properties, he proves by the following facts: 1st. That diseases analogous to those fevers have been induced by injecting putrid matters into the veins of animals: 2d. Animal poisons introduced into the blood easily produce these fevers, also in small pox, measles, and, 3d, These poisons gain access to the blood through the medium of the air, and by the lungs: 4th. The non-contageous fevers, as Remittents, and Intermittents depend upen a poisoned or changed state of the atmosphere: 5th. Observation shows that, in Essential or Idiopathic fevers, the blood is altered.

Not only is there toxœmia or blood poisoning, but that it is primarily so, the difference in the phenomena is owing to the difference in the character of that poison that each specific miasm has its specific effects. Urea in the blood has its legitimate uramic developments because it poisons the nervous centres, the virus of Small Pox attacks the skin and the mucous tissue, and yet the peculiar nature of each poison is not yet understood. Experience has perceived the legitimate action of each and a uniformity in the results show a similarity in the causes producing them. And yet, although these general relations obtain, there is not always an identity, for in Typhoid fever and in Small Pox the fibrine of the blood is defective.

As we have no certainty as to the nature of the impression, neither have we an assurance as to the peculiar therapeutic action of remedies. Mercury produces inflammation of the salivary glands, Arsenic the mucous tissues, Beladonna the skin, Ergot, the uterus &c.

*Zymosis* or fermentation of the blood, has recently received much attention, particulary at the hands of the illustrious Liebig. It is made the basis of a plausible hypothesis, long since advocated, but subsequently discarded. Its connection with Humoral Pathology attainted it, and it fell into disrepute and neglect. Liebig, in his work on "Animal Chemistry" advances the following position which is quoted and highly commended by the anthor of the Essay.

"The Chemical force and the vital principle hold each other in such perfect equilibrium, that every disturbance, however trifling, or from whatever cause it may proceed, effects a change in the blood." The Zymotic change then is due to a molecule in a state of dissolution within the body; this by contact diseases another, and so on, until the whole may may be diseased, and, this act of dissolution is termed "decomposition by contact," or the "action of presence."

Those bodies which contain *nitrogen* suffer most, and suffer first, and as a large share of this element is formed in the blood, therefore, dissolution once commenced, unless arrested, will speedily and certainly pervade the whole fluid mass. When this is not the case, then we must look for some sustaining principle, and this is found in the vital forces. Here, then, is the struggle. On the one hand is the vital law, asserting its powers and prerogatives; while on the other, is this Zymotic distemper, going on from molecule to molecule. Which shall triumph, will determine the fortunate or unfortunate result of the case.

But what proofs of this law of Zymosis? What are the proofs to show that putrefactive matters, by the entering into the blood, will produce the phenomena of fever? The answer is, that the putrid particles in the air of the dissecting room, communicated to the living body through the lungs, and putrid blood, brain, and eggs, laid upon recent wounds with an abrasion of surface have caused vomiting, and even death. Putrid matter injected into the blood of healthy animals, and a small portion accidentally introduced during dissection, will produce symptoms of Tyhus. Yeast or sugar, thrown into the circulation, will also produce typhoid symptoms with ecchymosis of the skin, and the decomposition of animal and vegetable matters in quantities, will produce epidemic diseases. The case of the Sexton, while in the act of lowering a coffin let it fall and the corpse being in a state of active decomposition, a large amount of sanies, highly offensive, was exposed and flowed out. One hnndred and fourteen persons present fell dangerously ill of a putrid disease. It spread subsequently to some sixty others with like results. The case of the man, who became diseased by skinning a diseased animal, sickened, and some of his blood injected into the groin among the lymphatics of a cat, produced fatal symptoms and it died in seven hours. These proofs are given to show that a poison may be introduced into the system, producing its legitimate phenomena, and when introduced into others and perpetuated carries with it, as resulting phenomena, similar manifestations and symptoms. The proofs, we regard as conclusive of the position taken and are familiar to every physician of experience.

The blood then is a great pool, a cesspool oftimes in certain forms of malignant diseases, in whatever way or manner generated.

The blood has been found in a partial state of dissolution in putrid diseases, is a fact made notorious by clinical observation, and we find under this condition the fibrine suffers.

The essayist accounts for the alkaline reaction in the urine, as the result of the presence of hydro-sulphate of ammonia in malignant diseases, and is the product of decomposition.

The importance of the depuraors, from the skin, the bowels, from the kidneys, is strongly urged, and yet not *too* much enforced. In a practical point of view, we think this one of the most interesting and useful aspects of this essay. One reason, the author assigns and which has been repeatedly maintained, why these functions should be stimulated to increased activity, is that if the morbific matters be not thrown off a "backward movement" obtains, the poison accumulates and all the symptoms are aggravated. The experiments of Orfila and of Golding Bird are referred to in order to show the importance of awakening the kidneys particularly, to increased depurative action.

He takes a conservative position with regard to the Humoralists and the Solidists. He adopts neither system wholly, nor yet does he entirely discard either. So long as the tissues look to the blood for life, and as long as the tissues undergo changes as a sequence of an altered condition of the blood, *so long* both are right. The one is a cause, and the other the effect of that cause.

The febrile state is the result of the alteration; the biotic forces are awakened because the nervous centres depend for their powers upon the integrity of the blood. This accounts for the great depression of animal and organic functions, to this state congestions follow, either with suspended or imperfect secretions, and there is a general languor of all the functions in these fevers.

The following important indications follow: 1st. To counteract the injurious operation of the poisons. 2d. To expel them from the system. The first is by the administration of salines, as Chloride of Sodium and the Chlorate of Potass, and by acids, as the hydrochloric, &c., &c. 2d. Tonics, stimulants, and depurants, alcohol and arsenic, are valuable antiseptics, because of the dissolved condition of the blood. (In the low putrescent forms creasote with chlorines?)

Fever is but the evidence that nature is endeavoring to effect a cure by throwing off the offending matters. This act is called an effort of the "vis medicatrix," and in the successful recuperative act, nature becomes the best and wisest physician.

This pathology reconciles the different modifications of diseased conditions under *epidemic influences.* We recognise two elements: *first,* the depressing poison of Zymotic state, and *second,* the inflammatory or functional derangements of local organs. This is nothing else than those conditions termed *sthenic* and *asthenic.* The treatment would be more rational. Blood letting and the antiphlogistic remedies in the latter must be used, if resorted to at all, most sparingly and with caution. The closing remark of the author we quote in his own language: "*The great success in the treatment of disease is to be found in a broad, comprehensive, and rational pathology—a pathology that weighs every element of disease, whether of fluids or of solids, that is capable of exciting, depressing or perverting vital actions.*"

We have at some length endeavored, but imperfectly however, to give the main leading points of this invaluable essay, that the reader may be able to understand somewhat its design and appreciate the importance of this contribution to medical science. We are aware that any attempt at a synopsis would be likely to detract from its true merits, and yet we hope it will be well received by all. The motive was just, and if we have failed, it was because we had not room to publish it entire. In the condensed extract we have occupied some pages to the exclusion of other matter, but we think not of more importance.

Our time cannot be better or more profitably employed than in the discussion of these grave questions connected with patbological science. The physiologist, aided by the microscope, and the chemist with his laboratory, are astounding the medical world with their discoveries, and making medical science radiant with new and brilliant revelations. It makes the soul within us to leap with joy, with the prospects of a still brighter and more glorious future in medicine, and that humanity will proudly exult in its increased attributes and triumphs. Every year adds fresh laurels to those which now bedeck her brow, and new jewels are being added to glitter in her coronet and crown. Within a few short years the untiring efforts of the medical philosopher have resulted in glorious and enduring results. Enduring, we say, because, in the main, the discoveries are *facts,* not suppositions; *truths,* not doubtful hypotheses. Microscopic investigations and chemical analyses, when successful in their experiments, produce results invariable and uniform, because the discovery of carb ammonia, of urea, &c., in the blood, and albumen in the urine, are products of which there exists not any longer doubt. These are refinements, it is true, upon former investigations, and yet they are mathematical results. The particular mode by which these abnormal changes are effected may remain obscured, as they are now measurably hidden from us, but doubtless we shall be able hereafter to detect other mal-products and trace out certain phenomena as the result of their presence with comparative certainty. The present age in medicine is not engaged in inventing new hypotheses or false theories, but it is proceeding practically into things substantial, tangible and real. Hereafter there will be less mist or fog found enveloping medical science; no longer expansive systems or dogmas invented by visionary speculators, to confuse and bewilder; and to which their followers, as in olden time, will cling as to a confession of faith; but the scalpel, the microscope, and the labratory are active in a utilitarian search after tangible objects and materials.

We shall at some future time call the attention of readers to the consideration of some features of this valuable treatise, with the view of keeping the subject before the medical public. Here, where we have yearly visitations of the enieric forms of fever, the discussion of these subjects is of grave importance, The pathology of these fevers cannot be too much studied and examined in order to arrive at the most rational and effective treatment. EDITORS.

